# 926 Specialist Within a Specialty: Demystifying the Role of the Burn Nurse Specialist

**DOI:** 10.1093/jbcr/iraf019.457

**Published:** 2025-04-01

**Authors:** Stacey Richerbach, Tiffany Hockenberry, Karen Richey, Kevin Foster

**Affiliations:** Diane & Bruce Halle Arizona Burn Center; Diane & Bruce Halle Arizona Burn Center; Diane & Bruce Halle Arizona Burn Center; Diane & Bruce Halle Arizona Burn Center

## Abstract

**Introduction:**

The ever-changing landscape of modern burn care is associated with an ever-evolving role of nurses in the specialty. Nurses are integral to the advancement of healthcare as leaders, researchers, and educators. One role that has become increasingly prominent is the Burn Nurse Specialist (BNS). References to the BNS date back to 1987, yet published information is scarce. In 2019, it was recognized that there was an unmet need for both bedside quality improvement initiatives and real-time education in our center, leading to creation of the BNS position. This review aims to demystify the function of the burn nurse specialist and identify challenges in implementation.

**Methods:**

This qualitative review of the BNS in our center examined job creation, responsibilities, challenges, and evolution of the role. Within the center, two nurses have been employed in the role. Personal accounts and perspectives of both were ascertained.

**Results:**

The BNS role was added without a foundation for duties, expectations, nor training resources. High level functions noted in the job description were expansive and included “clinical nursing practice, education, consultation, performance improvement, supervision of staff and staff development.” The BNS was to lead, serve as a resource for daily operations, and ensure alignment with organizational goals and leadership. Requirements included both clinical and supervisory experience, with a master’s degree or higher preferred. Challenges included role development, training, and compensation determination. Specifically, there was no foundation for daily operations and as the only BNS in the state, calculation of compensation was problematic. The role was retitled to an existing position, Clinical Nurse Support Specialist, for comparison. The role remained in the Burn cost center. The job has evolved to encompass quality improvement (QI), interdisciplinary program and protocol development, close collaboration with the nurse educator and research team, serving as a liaison between the bedside and support services. Data-driven recommendations are used to remediate institutional barriers and challenges. Action plans and resources developed through this role have been widely adopted throughout the organization.

**Conclusions:**

By maintaining a finger on the pulse of patient care, the BNS informs strategies for optimizing outcomes, bringing evidence-based practice to the bedside. Daily responsibilities are variable as the BNS must readily adapt to address actual and anticipated center needs. The BNS is a valuable resource for patient care, protocolizing evidence-based care, and facilitating loop-closure for the team.

**Applicability of Research to Practice:**

This review provides insight for other centers considering the BNS role.

**Funding for the Study:**

N/A

